# Pervasive antibiotic misuse in the Cambodian community: antibiotic-seeking behaviour with unrestricted access

**DOI:** 10.1186/s13756-017-0187-y

**Published:** 2017-03-24

**Authors:** Chhorvoin Om, Frances Daily, Erika Vlieghe, James C. McLaughlin, Mary-Louise McLaws

**Affiliations:** 1 School of Public Health and Community Medicine, UNSW Medicine, UNSW, Level 3 Samuels Building, Sydney, 2052 NSW Australia; 2 Diagnostic Microbiology Development Program, # 23 (First Floor), Street 310, BKK 1, Khan Chamcar Morn, Phnom Penh, Cambodia; 3 Department of Clinical Sciences, Institute of Tropical Medicine, Nationalestraat 155, 2000 Antwerp, Belgium

**Keywords:** Antibiotic resistance, Behaviour, Unofficial prescriber, Self-medication, Nurse prescriber, Pharmacy, Drug outlet

## Abstract

**Background:**

Antibiotic misuse is widespread in resource-limited countries such as Cambodia where the burden of infectious diseases is high and access to antibiotics is unrestricted. We explored healthcare seeking behaviour related to obtaining antibiotics and drivers of antibiotic misuse in the Cambodian community.

**Methods:**

In-depth interviews were held with family members of patients being admitted in hospitals and private pharmacies termed pharmacy attendants in the catchment areas of the hospitals. Nurses who run community primary healthcare centres located within the hospital catchment areas were invited to attend focus group discussions. Nvivo version 10 was used to code and manage thematic data analysis.

**Results:**

We conducted individual interviews with 35 family members, 7 untrained pharmacy attendants and 3 trained pharmacists and 6 focus group discussions with 30 nurses. Self-medication with a drug-cocktail was widespread and included broad-spectrum antibiotics for mild illness. Unrestricted access to antibiotics was facilitated by various community enablers including pharmacies or drug outlets, nurse suppliers and unofficial village medical providers referred to as “village Pett” whose healthcare training has historically been in the field and not at university. These enablers supplied the community with various types of antibiotics including broad spectrum fluoroquinolones and cephalosporins. When treatment was perceived to be ineffective patients would prescriber-shop various suppliers who would unfailingly provide them with antibiotics. The main driver of the community’s demand for antibiotics was a mistaken belief in the benefits of antibiotics for a common cold, high temperature, pain, malaria and ‘Roleak’ which includes a broad catch-all for perceived inflammatory conditions. For severe illnesses, patients would attend a community healthcare centre, hospital, or when their finances permitted, a private prescriber.

**Conclusions:**

Pervasive antibiotic misuse was driven by a habitual supplier-seeking behaviour that was enabled by unrestricted access and misconceptions about antibiotics for mild illnesses. Unofficial suppliers must be stopped by supporting existing regulations with tough new laws aimed at outlawing supplies outside registered pharmacies and fining registered pharmacist/owners of these pharmacies for supplying antibiotics without a prescription. Community primary healthcare centres must be strengthened to become the frontline antibiotic prescribers in the community thereby enabling the community’s access to inexpensive and appropriate healthcare. Community-based education program should target appropriate health-seeking pathways and the serious consequences of antibiotic misuse.

## Background

Inappropriate antibiotic use is a global problem and contributes to the global crisis of antibiotic resistance [1, 2]. Global antibiotic resistance severely affects developing countries where infectious diseases are endemic [3, 4] and persistent inappropriate antibiotic use prevails [5–8]. Infectious diseases cause 58.6% of deaths and 63.6% of disability-adjusted life years (DALY) loss for the global poor which is in stark contrast in the global richer countries where deaths and DALY loss from infectious diseases account for just 7.7% and 10.9%, respectively [9]. A study of antibiotic use in 12 low-resourced countries found between 25 and 75% of patients received antibiotics inappropriately from primary healthcare [10]. Global community surveys reported that between 19 and 100% of antimicrobial use outside northern Europe and North America was non-prescribed [7].

In developing countries inappropriate antibiotic practice involves both the patient and the healthcare provider and is driven by the patient’s socio-economic level and behavioural and cultural factors of both the patient and practitioner [8, 11–14]. Other contributing factors of inappropriate antibiotic use in low-resourced settings include cultural healthcare seeking preferences, beliefs that antibiotics are magic and strong drugs can cure and prevent many diseases [13]. A study in neighbouring Vietnam reported that mothers received antibiotics from community privately owned unofficial drug outlets for anticipated symptoms such as sore throat, cough, fever and diarrhoea [15].

Cambodia is not immune to multidrug-resistant microorganisms that cause common infections, such as bloodstream and urinary tract infections, in various locations around the country [16–19]. Not surprising, extended spectrum beta-lactamase resistance was found in 48% of *E. coli* isolates from adult bloodstream infection in Cambodia while the rate of methicillin-resistance *Staphylococcus aureus* was also high at 22% of *S. aureus* isolates [19]. Inappropriate prescribing is also common in Cambodia with estimates between 60 and 100% of all visits to primary healthcare providers receiving an antibiotic [20, 21]. Non-prescription antibiotic use is a common practice in the Cambodian community [22, 23] with respiratory tract infections and diarrhoea in children under five years of age being the most common indication for antibiotic use [23]. There are no studies that have explored the antibiotic-seeking behaviour in the community and we report here an exploration of this behaviour and drivers of antibiotic misuse in Cambodia.

## Methods

### Study design and setting

We used qualitative in-depth interviews with individuals and focus group discussions (FGDs) with nurses from primary healthcare centres to collect data between September 2013 and February 2014. Cambodia is a lower middle-income country located in Southeast Asia with a population in 2015 of 15.58 million [24] of whom 80% reside in rural areas [25]. Although poverty has declined it still prevails at a rate of 17.7% with 8.1 million Cambodians described as poor or near poor [24]. The majority (90%) of the Cambodian poor live in the countryside [24]. The Cambodian public healthcare system consists of 1236 healthcare facilities including 91 hospitals, 1024 healthcare centres that charge very low fees and 121 remote rural healthcare outposts that are staffed twice monthly [26]. Over half of all healthcare services are provided by the private sector [27].

### Sampling and data collection

A purposeful sampling method [28] was used to select a sample of public hospitals for the participation in a cross-sectional survey of physicians’ prescribing prior to this study [29]. A purposeful selection of family members of patients admitted to the selected public hospitals was used to enrol participants for individual interviews. Purposeful selection was used for pharmacy attendants from private pharmacies and nurses from community primary healthcare centres located within the catchment areas of the selected hospitals for interviews and FGDs, respectively. A Cambodian physician (CO) conducted all interviews and FGDs in Khmer. Data collection continued until data saturation was reached [30, 31]. Informed consent was collected from each participant and all interviews and FGDs were digitally recorded.

### Data analysis

All digital audio records of interviews and FGDs were transcribed verbatim into Khmer language. The transcribed Khmer texts were checked and edited against the digital audio records for accuracy before being translated into English by a local translation consultant. All the English translations were checked and edited by two independent consultants: the physician who edited the Khmer text transcription and an independent English-Khmer speaking consultant who conducted overall checks of the translation. One author (CO) checked the translation against the transcribed Khmer text and edited any portions that were unclear. Data were analysed using thematic data analysis and presented as thematic syntheses [32, 33]. Data were inductively coded by two researchers separately (CO and MM) to insure validity [34]. Data were coded using Nvivo version 10 software program while another coder coded the text directly. The two coders compared the coding and checked for consistency and any discrepancies were addressed.

## Results

We conducted 45 individual interviews with 35 family members of patients being hospitalized and 7 untrained pharmacy attendants and 3 trained pharmacists. Six FGDs were conducted with 30 nurses from 6 community primary healthcare centres within the catchment areas of the hospitals where family members were interviewed. The findings revealed a widespread misuse of antibiotics in the community that was facilitated by antibiotic-seeking behaviours, unrestricted access to antibiotics, and poor knowledge about antibiotics.

### Healthcare and antibiotic-seeking behaviour

Participating family members spoke about the severity of illness and the factors that influenced their healthcare and antibiotic-seeking behaviour. Judgement of the severity of symptoms was a common practice used to determine what level of healthcare would be accessed. For a mild illness such as a common cold, mild diarrhoea or general unwell feeling patients would self-prescribe medication purchased and freely available from a nearby pharmacy or village drug outlet.

*“When I have a minor headache, sore throat or fever I go to buy medicines from pharmacy.” Family Member # 2-NMCH*

*“When I’m sick such as from headache, tiny ache, I always buy medicines in the village; 1 dose, 2 doses [something] like that.” Family Member # 2-BT*

In local terminology the words *Dambao Krapeas* (gastric ulcer), *Roleak Krapeas* (gastritis), *Dambao Posvean* (intestinal ulcer), or *roleak Posvean* (intestinitis) all refer to internal inflammation or wound. Because the community uses antibiotics for external wounds, injuries and inflammation the family will seek antibiotics to heal all ‘internal’ pain or illnesses they perceive to be caused by inflammation or internal ‘wound’.

*“I usually take one para [paracetamol] and one ampi [ampicillin]. Then I don’t feel any more pain in my stomach.” Family Member #3-NPH*

*“Phsas (healing) in case we have inflammation, intestinal inflammation, gastric inflammation, other wound we can take ampi [ampicillin].” Family Member #4-KCH*

After having taken a cocktail of drugs and when family members perceived that symptoms were unrelenting and severe such unendurable pain, colic or fever they would attend a community primary healthcare centre or hospital. If their finances permitted they would visit a private healthcare provider.

*“In case of minor sickness, I go to buy medicines but I go to a healthcare centre if it is a severe illness.” Family Member # 2-KRV*

*“I go to hospital when I have a sharp pain in my stomach and/or bowel....like when there is an inside pain, which we cannot know what it is. I cannot go to a pharmacy.” Family Member # 7-BT*

*“We always go to a [private] clinic if we have a severe sickness.” Family Member # 10-MBR*

These patients will also consult several healthcare providers when they perceive their treatment has been ineffective including unofficial healthcare providers known in the community as village *Petts*. The community ‘prescriber-shopper’ would request after-hours private care from physicians, nurses or village Petts.

- *Interviewer: Did you go to physician H; how many consultations? 5 consultations?*
- *Participant: We visited physician H 3 times. He gave medicines and my husband got better. After he was better, he relapsed.*
- *Interviewer: Then you went to another healthcare provider?*
- *Participant: Yes, after thinking that the hospital was [too] far we went to Pett K.*
- *Interviewer: How many times did you consult Pett K?*
- *Participant: Two times. He injected [my husband] and he gave medicines. When we came the third time he told us that he couldn’t treat [him]; couldn’t continue any more. He told us to come to this hospital.*

Healthcare providers are often referred to as Pett and this term includes unofficial “village Pett”. The unofficial village Petts were healthcare providers during the Khmer Rouge genocide period (between 1975 and 1979) or unofficially trained in the Cambodia-Thai border camps during civil unrest after the Khmer Rouge period, and those who have unknown medical training history. These unofficial suppliers have remained practitioners in the community making house calls where they provide their patients with a diagnosis and treatment that includes antibiotics.

- *Interview of Family Member # 001_A_002 KP*
  - *Interviewer: Who is that private Pett? Is he a doctor? Where does he work?*
  - *Participant: I heard that he is a military Pett coming from the camp [Cambodian-Thai camp during civil unrest].*
- *Interview of Family Member #4-KCH*
  - *Participant: I fetched a Pett to give treatment at my home, the Pett in the village.*
  - *Interviewer: Village Pett … is he a staff from a health centre?*
  - *Participant: He is not a staff in a health centre. He just gives treatment to villagers; but he is not a health centre staff.*

Despite perceptions of the severity of an illness that influences healthcare-seeking behaviour, family members described other factors that include:

- Convenience
*“I generally go to the healthcare centre near my house. I only pay 1000 riels and I’m given medicines for three days.” Family Member # 3-KTL*
- Burden of balancing demands of earning an income, taking care of children, the elderly and their properties at home, with providing basic care in the hospital to their family member during their admission;
*“I have small grandchildren, their parents go to work. I said to the hospital staff ‘if my husband has typhoid fever please give him IV injections at home’. I don’t want to go to hospital because there’s no one to take care of him in hospital as I’m at home with the grandchildren.” Family Member # 1-KRV*
- Ability to pay healthcare providers and purchase of medicine.
*“If I have money I buy medicines, to take two doses. But if I don’t have money I can only buy medicines to take once only.” Family Member # 4-KTL*
- Trust in the safety of the healthcare provider and effectiveness of the treatment;
*“It is safer to come to this hospital than to buy medicines outside. We meet the doctor and he advised us to take this or that [medicine] which is effective.” Family Member # 8-MBR*

Nurses from primary healthcare centres and a trained pharmacist at a provincial pharmacy explained how the financial constraints of the patients prevented them from initially consulting a physician and how this drives poor medication behaviour:

*“In real practice as I have experienced the patients just let us inject for one or two days. If they are better, they stop because they don’t have the money [to continue treatment].” Nurse #1-SLL5*

*“For minor cases mostly patients come to buy one or two doses of medicines at the pharmacy because they don’t have much money. They do this because they don’t have money to see a physician.” Pharmacist # 2-SRP*

### Unrestricted access to antibiotics

Access to antibiotics is unrestricted and facilitated by various community enablers who include trained private pharmacists and untrained pharmacy attendants, drug outlet suppliers, unofficial village Pett suppliers, nurses and trained physicians. The community is so familiar with the antibiotics that are freely available that they know the name of many such as pen [penicillin], amox [amoxicillin], ampi [ampicillin], tetra [tetracycline] and cotrim [co-trimoxazole]. These antibiotics are even freely available in small village grocery shops in remote regions.

*“When I have a high temperature or a cold, amox [amoxicillin] and ampi [ampicillin] are given to me mixed together. If I have a runny nose the drug sellers give me ampi, and if this isn’t available it’s replaced with amox.” Family Member #2-NPH*

*“After I realize that cotrim [cotrimoxazole] can cure diarrhoea I always buy it. I buy only one tablet and I crush and grind it with water for my children to drink.” Family Member #1-SRP*

Most drug sellers in pharmacies or drugs outlets were untrained. But training does not always guarantee appropriate antibiotic use. When asked when antibiotics were indicated both trained pharmacists and untrained suppliers believed that antibiotics were indicated when their patients had diarrhoea and respiratory symptoms such as sore throat, fever and cough. Both trained and untrained suppliers drive the antibiotic-seeking behavior by providing a cocktail of drugs based on symptoms and this cocktail includes non-prescription medications plus an antibiotic such as amoxicillin, ampicillin, cephalexin, cefixime or co-amoxyclavulanic acid for upper respiratory infections and co-trimoxazole for diarrhoea.

*“For normal colds without high temperature, patients can take amoxicillin. If they cough or have a high temperature, they take augmentin or cefixime. It depends on the seriousness of the sickness.” Untrained pharmacy attendant #1-PP*

*“It is like what I told you earlier. Antibiotics are mostly used in case of diarrhoea, high temperature, cold and cough.” Untrained pharmacy attendant # 1-NL*

*“Antibiotics should be prescribed for sicknesses such as respiratory inflammation.” Untrained pharmacy attendant #1-KCH*

*“As my routine, I listen to their [patient’s] sickness first. If they have a headache, then I give Alaxan [ibuprofen and paracetamol] or Para [paracetamol]; if they have a cold, I give them Decolgen [anti allergic, decongestant and paracetamol] and vitamin. I can use amox or ampi in the case that they have a sore throat or have a fever for many days ago.” Trained pharmacist #2-SRP*

Nurses from public hospitals and community primary healthcare centres commonly provide private after-hours services that offer a diagnosis and treatment. When asked “Have you ever invited nurses to your house for treatment?” family members indicated they provide injections and antibiotics:

*“Yes. It [occurs] in the case [when] we are very busy at home and don’t have enough time to get treatment at [a] clinic, then we can call the nurse to treat us at our house.” Family Member #9-MBR*

*“He (nurse) told me that I had gastric inflammation and then he wrote ceftri [ceftriaxone] injection on a piece of paper. He asked me ‘do you want drugs at the healthcare centre or want me to give the injection at home.’ I thought I wanted him to give the injection [at home] because I don’t have time to go to the healthcare centre to take drugs.” Family Member #6-KCH*

Nurses who are employed in primary healthcare centres also have a private practice and prescribe fluoroquinolones and 3^rd^ generation cephalosporin and often provide injections.

*“If it’s typhoid fever we prescribe oflocet [fluoroquinolones] for 5 days. If they [patients] don’t recover we prescribe ceftri [cetriaxone].” Nurse #3-NL*

*“If the sickness becomes more severe, we have to use IV injection and ceftri [cetriaxone]. If the sickness is just a simple fever, which is not serious, we continue using oflocet [fluoroquinolone] for 3-5 days.” Nurse #2-NL*

Unofficial village Petts are unregistered or have undefined training and supply medications and antibiotics including broad-spectrum antibiotics in the community.

*“Village Pett are Pett from Pol Pot regime [Khmer Rouge] who have experience (providing health care during that time). They sell medicines and if we need ampi [ampicillin] or amox [amoxicillin], they will sell them to us. They provide injections too.” Family Member #3-BT*

*“It is the same for both treatment at the healthcare centre and treatment from door to door. Some people use Amox as Thnam Psas (antibiotic). Village Petts providing treatment from door to door mostly prescribe Ceftri [ceftriaxone]. They prescribe the high ones. Ceftri is generally used.” Nurse #2-NL*

Official healthcare providers were concerned about these village Petts providing care and antibiotics but did not recognise their own professions’ role in misusing antibiotics.

*“Yes, there are [unofficial Petts] but far from here in the remote rural area. There are also some Petts who just learn from each other and never went to school. Now we still cannot stop this problem.” Nurse # 1-SLL5*

### Poor knowledge about antibiotics

Family members were asked about the benefit of antibiotics with the interviewer who used the local term *Thnam Phsas* or the antibiotics by name such as amoxicillin, ampicillin or penicillin. They described how *Thnam Phsas* could heal minor wounds (‘dambao’), injuries (‘robuos’) or cuts (‘mout’) and inflammation (‘roleak’) such as throat inflammation (‘roleak bampongkor’).

*“If there is a wound [dambao], mostly people take Thnam Phsas. It can heal both inside and outside of the body.” Family Member #8-MBR*

*“We take ampi [ampicillin] when we have cuts [mout] on arms or legs or when we have a motorbike accident or other small accident. When we take it [ampicillin], it helps to heal the injury [robuos].” Family Member # 2-NMCH*

*“If we have cough and we’re afraid that it will cause throat inflammation we take Thnam Phsas. When I tell drug sellers that I have a cough, cold or sore throat, then they will cocktail it [Thnam Phsas] for me.” Family Member #13-SRP*

*“Based on my understanding, Thnam Phsas [antibiotic] is for throat inflammation.” Family Member #4-NL*

Trained pharmacists, untrained pharmacy attendants and nurses from community primary healthcare centers upheld the community’s belief about the benefits of antibiotics that they use to treat inflammation.

*“Generally drug cocktail is for common cold, cough or mild sore throat. For common cold and cough I don’t give antibiotic in the cocktail. I only give antibiotics for throat inflammation.” Trained pharmacist #001_A_007 PP*

*“Antibiotics should be prescribed for sicknesses such as respiratory inflammation.” Untrained pharmacy Attendant #3-KCH*

*“For those who have minor sicknesses, sometimes I do not prescribe antibiotics for them, never. Mostly I prescribe them as long as they have throat inflammation. It is like this.” Untrained pharmacy attendant #3-NL*

*“If we see that they have upper breathing inflammation or bronchitis, we prescribe antibiotics for them [patients].” Nurse #5-KRV*

*“We think that the disease could be something related to inflammation, and then we use antibiotics.” Nurse #3-SLL5*

Besides a belief that antibiotics were beneficial for injuries, wounds and inflammation, family members described further misconceptions that antibiotics could be beneficial for the common cold, high temperature, pain and even malaria.

*“Ampi [ampicillin] and amox [amoxicillin] help us, for example, when I have a stuffy nose, it helps to reduce the clogging and I can breathe more easily, feel better, reduces the temperature and so on.” Family Member #2-NPH*

*“When I have pain, we take it [amoxicillin]. It helps to reduce pain.” Family Member #2-KRV*

*“Yes. Ampi [ampicillin] for less severe disease has less phsas (healing) substance but it has cooling substance inside. For malaria, we can take it [ampicillin] in a mixture as well. For ampi [ampicillin], if there is amox [amoxicillin], we still use ampi [ampicillin] because it has cooling substance. I use [it] like this.” Family Member #4-KCH*

## Discussion

Misuse of antibiotics was widespread in the community that was driven by a determined antibiotic-seeking behaviour facilitated by both trained and untrained healthcare providers who supplied and enabled the community’s antibiotic use. All actors held poor knowledge about the indications and uses of antibiotics: the community, trained pharmacists and unofficial suppliers who include untrained pharmacy attendants, drug outlets, nurses and village Petts. The source of the unrestricted access to antibiotics was pharmacies and drug outlets. Through these sources self-medication with antibiotics and drug-cocktails has become a community norm that may expedite antibiotic resistance [7]. These sources also provided nurses and village Petts with easy access to antibiotics that they would supply the community during house-calls.

The services provided by village Petts are unsafe because most would have received training without examinations to ensure that they had reached a minimum acceptable level of medical knowledge. In 2014, an unofficial village Pett was responsible for an outbreak of HIV infections among Cambodian villagers from unsafe injecting practices [35]. In November 2016 a new law was passed requiring all healthcare professionals to register with their relevant professional body for licensing. At the same time new penalties of up to 2 years jail were legislated that outlawed unregistered providers of diagnoses and treatments but it is too early to determine whether the legislation will be success. To date, the current implementation of laws and regulations pertaining to the registration of trained pharmacists is not working [36, 37] to stem the flow of non-prescription antibiotics. Methods of improving the effective enforcement of existing pharmaceutical law and regulation need to be developed that would prevent antibiotics from being sold outside registered pharmacies. Improved regulations should carry fines for pharmacies that do not have a trained pharmacist on the premises during trading hours and fines where antibiotic usage does not match prescription numbers.

**Fig. 1:**
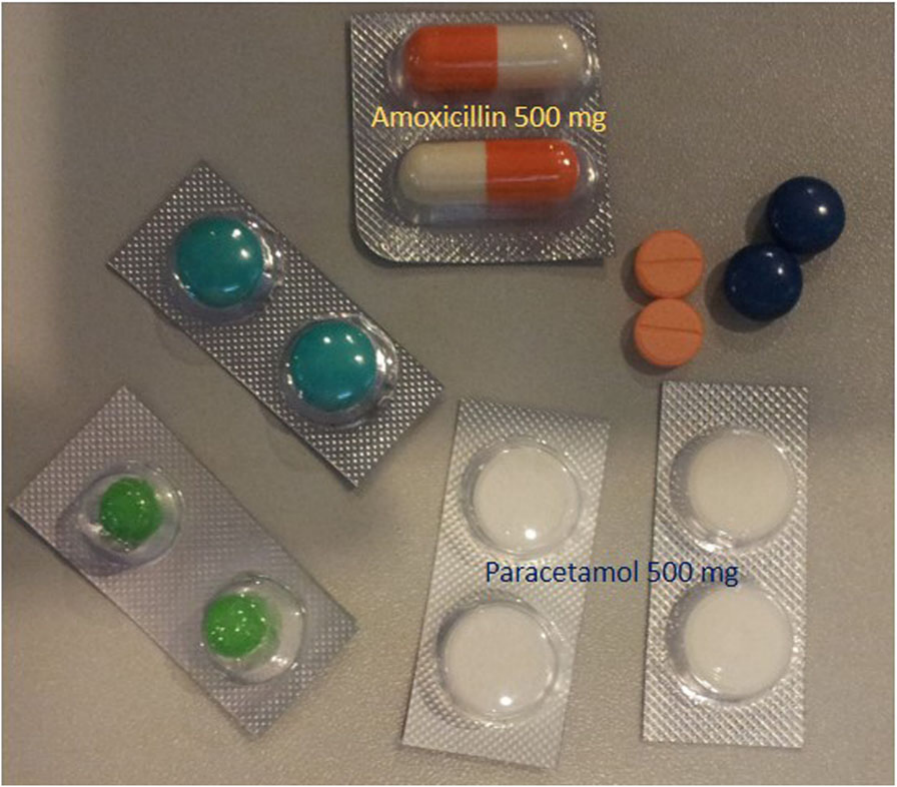
Drug cocktails for common cold purchased from a village drug outlet

Limited household finances impede safe healthcare-seeking behaviour in developing countries [8, 12, 13] and this was an important barrier for our community. Poor understanding about the importance of the correct indication for and dosage of antibiotics in our community has aided supplier-shopping and in other communities [8, 38]. Although poor knowledge facilitated the practice of purchasing one or two doses of antibiotics neither the pharmacists nor the home-visiting nurses corrected this practice. There are an estimated 13,000 village drug outlets across Cambodia [23] that provide antibiotics over-the-counter without a prescription. During our fieldwork we sent in a mystery shopper who easily purchased a cocktail of drugs for his common cold symptoms. Our mystery shopper was given two doses of 500 mg amoxicillin and several unknown medications for his symptoms (Fig. 1). Apart from the misuse of antibiotic for the common cold [39] one or two doses of amoxicillin may cause resistance in commensal bacteria [40, 41]. One or two doses of antibiotics is insufficient treatment for the majority of bacterial infections. As an example the treatment of amoxicillin sensitive group A β-hemolytic streptococcal pharyngitis is 1000 mg of amoxicillin once-daily for 10 days [42]. In addition to the contribution by drug outlets to inappropriate dispensing of antibiotics the drug outlets also flood the community with counterfeit and substandard antibiotics [43]. Counterfeit antibiotics place pathogens under pressure to develop antibiotic resistance and if antibiotics are eventually prescribed appropriately for patients who has recently used counterfeit antibiotics the treatment may be ineffective [44]. The Cambodian Ministry of Health enforced the closure in 2010 of nearly 65% of illegal pharmacies across the country [37] but the implementation of these laws and regulation remains weak [45]. The community’s expressed appreciation of trained healthcare providers for a serious illness may help the success of the 2016 legislation of only registered physicians to provide treatment, but only if there was an unrelenting public education campaign that runs in conjunction with fines for unlawful antibiotic supply.

## Conclusions

The magnitude of antibiotic misuse in the Cambodian community was driven by antibiotic-seeking behaviour that would not be rewarded without unrestricted access to antibiotics. The misconceptions of the indications for antibiotics would have less impact on antibiotic-seeking behaviour if unfettered access to antibiotics were prevented. The control of antibiotic access requires the immediate outlawing of antibiotic access from source other than a registered pharmacist and a prescription. This would also prevent the direct access to antibiotics by nurses and village Petts who supply antibiotics during home visits. Educational programs about the serious consequences of antibiotic misuse could be dealt with by providing appropriate and affordable healthcare-seeking pathways to become the first point-of-care for strictly regulated antibiotic prescribing.

## Abbreviations

DALY: Disability-adjusted life year
FGD: Focus group discussion
UNSW: University of New South Wales
WHO: World Health Organization
